# Odontological Society of Pennsylvania

**Published:** 1893-08

**Authors:** 


### ODONTOLOGICAL SOCIETY OF PENNSYLVANIA.

The regular meeting was held June 10, 1893. Vice-President Dr. C. R. Jefferis in the chair.

Before the question of the evening was taken up for discussion, Dr. Truman exhibited the Morgan-Maxfield disk-mandrel, as many present were not familiar with it. Its great simplicity, admitting of rapid changes in the disk, and firm retention constitute its principal advantage over some in the market.

Dr. Boice mentioned the case of a lady who had lately been treated by physicians for some lesion supposed to be connected with the ear, but which he demonstrated to have its origin in a wisdom-tooth. The pulp was exposed, and the application of sulphate of morphia allayed the pain. The pain in the ear was altogether of a reflex character.

The question of the evening was then taken up for consideration: "To what Extent and under what Conditions are the Collar and Crown the Cause of Pericemental Inflammation?"

Dr. Kirk opened the discussion. He said, "All subjects of this character present two phases. The first, the practical or utilitarian side, and, second, that of the philosophical or scientific, from which we deduce certain principles or rules for the application of the art. In that view, it seems to me, it will be better to invert the order of the question, and say, Under what conditions and to what extent is the collar-crown a cause of pericemental irritation?

"In the first place, the collar crown, as I understand it, includes all crowns of the Richmond type, and will also include any crown in which a collar is made to encircle the root. The only proper answer to the first part of the question seems to me to be, when it is an irritant, and that brings out the question of the conditions under which it may be an irritant. It may become such when the crown is improperly constructed. The accurate fit of a crown implies the proper preparation of the root and the proper preparation of the crown. The greatest source of failure in crown-work is when an attempt is made to fit a collar and crown to a root which is flaring at the free end. It is a mechanical impossibility to make a band fit a root in the mouth which has a flaring end. The flaring root-end with a band placed upon it produces a V-shaped gutter around the root which affords a lodging-place for food *débris*, and the margin of the gum becomes inflamed in consequence. There is a parallelism in the case of inflammation by reason of an irritating collar and inflammation about a tooth suffering from pyorrhœa alveolaris. The ragged edges and imperfect finish of the collar is an important item, but I think one of less importance than the first-named cause,—viz., imperfect adaptation of the collar to the flaring root-end. Drs. Black, Barrett, and some others hold that under certain systemic conditions irritation of the pericemental membrane produces a deposit of sanguinary tartar which becomes a local irritant. Both of them hold that such deposit may take place without the previous formation of a pus-pocket similarly to arthritic deposits in the tissues about a joint. This I never have been able to establish definitely; but we do have irritation at times from a band encircling the root, and subsequent conditions similar to those present wherever we have a deposit of sanguinary tartar.

From the irritation of the collar-crown we have a subacute or chronic form of inflammation on which from time to time develop periodical attacks of acute inflammation exactly similar to that which we have in typical forms of pyorrhœa.

"The question of the depth to which the band is forced under the gum is, I believe, of less importance than that of its adaptation. It is remarkable to what an extent a collar may be forced under the gum, if it accurately fits the root, if all the free edges are burnished down, without producing irritation. If it be a question of fit and depth of collar, it is important that this depth should be accurately gauged, and the fit should be carefully made.

"Another important point is the restoration of the anatomical contour of that portion of the root which has been removed. I present for your inspection two pieces which, to my mind, are ideal crowns in their construction, and a crown so made would not produce irritation to the gum. These are crowns figured in the *Dental Cosmos*, made by Dr. Shields, of New York. I have never seen any work that is so nearly perfect in its adaptation as that which he has made. His method is fully described in the *Dental Cosmos*, but as the illustrations do not show the remarkable beauty of the work, I brought these samples for inspection.

"The circumstances which are likely to produce irritation are those of imperfect fit and heavy, irregular edges. As to the extent of irritation it seems to me equally clear. It may go on until the tooth is completely lost by continued inflammatory action, the same as pyorrhœa removes the tooth from the socket."

*Dr. Deane.*—There seems to be nothing left unsaid by the former speaker on this subject, but I cannot agree fully with all of his remarks. I understood Dr. Kirk to say it was not material to him how far the band may be driven up on the neck of the root if it be a fit. I believe there is a limit to this, and you have reached that at the alveolus. To force a foreign body above this line will cause irritation, and through the different stages we reach suppuration. I have in mind a case which illustrates this. A lady called about a year ago with the left superior bicuspid capped with a gold crown very loose and the root very much inflamed. A discharge of healthy pus appeared on the distal margin of the gum, and the tooth was extremely sore to the touch. I succeeded in removing the crown, and as I have it here, you can examine it. The distal portion of the crown was left somewhat longer than the balance. I found, on examining the root, that this was done in order to cover a space destroyed by decay, that portion of the root being completely destroyed up to and above the alveolar line. After a few days' careful treatment the flow of pus had disappeared, the soreness left the root, and, much against the patient's will, I made an effort to restore the parts. I first set a post of wood into the root, extending down below the gum-margin, then with a thin matrix of platinum forced up the distal side of the root until it passed the alveolus. I formed a cavity for an amalgam filling, packing this with a quick-setting amalgam. I removed the matrix, withdrew the wooden plug, and I had restored the original contour of the root. A so-called Richmond crown was mounted. I saw the patient yesterday, a lapse of about one year's time since the operation, and she assures me she does not realize any trouble in the case at all. This and some others convince me that we must not band the roots too high. And again, this trouble is increased by allowing the surplus of phosphate of zinc to be forced out around the band rather than place a vent for it in the crown of the tooth; the latter method I believe to be preferable. To my mind, a band around the root of sufficient width to protect the root and keep it from splitting ought not to cause pericemental inflammation.

Dr. Kirk stated that Dr. Deane had misunderstood him; that he freely acknowledged that it was a source of irritation, but he thought less than that of an ill fit. He agreed with Dr. Deane that it should be simply butted up against the pericemental membrane.

*Dr. Truman.*—One expression in the remarks made this evening by several seems to me to imply a degree of ignorance, and should not be used, and that is, "Nature tolerates." According to my view, all operations of nature are governed by law, and is, therefore, universal in its application. There may be apparent exceptions, but it is our duty to discover the cause of these. Where there is apparent antagonism to the universal law of irritation, there is a reason for it not to be explained by the phrase, Nature tolerates it. Wherever there is roughness or space between the band and the root there will arise fermentation through the development of micro-organic life, and this will act upon the pericementum and finally end in its destruction. The objection that I have always held to banding teeth, while it has been modified in the course of years, still holds in regard to this treatment. As a rule, if a band is forced down on the pericementum, it is a reasonable inference that inflammation will follow. The pericementum is a delicate organ. Undue irritation will arouse inflammation. The symptoms are precisely the same, in my judgment, to those found in pyorrhœa alveolaris, and while this subject is not up for discussion, it can hardly be passed over, because the action of a band where it produces irritation simulates exactly that condition. You can have pyorrhœa produced by irritation, and that may proceed from a slight disturbance of the parts, and then extend to the pericementum. This may be produced by ligature or rubber dam. You have all noticed the bleeding of the gums upon the use of the brush. If you examine the gums you will find a red line on one or more of the teeth, and that line is simply the beginning of inflammation, and it will occur when a band is put on a tooth, unless it is perfectly made, and I question the possibility of making one absolutely perfect. The reason many do not produce irritation is simply the temporary effect of the phosphate of zinc; as long as that remains intact there may be no trouble, but as soon as the smooth surface gives place to the roughness, caused by acid action, the conditions are present for irritation.

*Dr. Woodward.*—I saw a case a few years ago, after the Richmond crown was first brought forward, in which a crown had been placed upon a bicuspid root. The root was one which tapered rapidly from the gum to the apex. In less than six weeks after it was put in the patient removed it with the thumb and forefinger, and with it came a piece of the alveolar process. This was the result of a band around the neck of the tooth.

My experience with the collar is that if it fits the root closely you will get very little irritation, but the gum invariably recedes from the collar. In time the band, which was once covered, will be found to be uncovered. In cases where the gum does not recede there is usually a low form of inflammatory action. I think if anything interferes with the attachment of the gum to the root, whether it be a piece of rubber or a ligature or a wedge or any substance, it will be followed sooner or later by recession of the gums. It naturally recedes as we grow older, and horse-jockeys take advantage of that fact when they want to buy or sell a horse.

Some years ago I filled two teeth for a gentleman, and had great difficulty in keeping my rubber above the cervical margins. Eight years afterwards the gum had receded to such an extent that I wondered that it had ever given me any trouble to keep the rubber dam on.

Dr. Boice, by drawings on the blackboard, described a case of a central incisor, broken off, and which he had built up for a patient. He first forced in cotton saturated with varnish, and used gutta-percha, wax, and chalk; coated it over until the gums would not bleed, and then proceeded to fit a brown or gold ferrule over the free margin, and inserted a post to hold it in place; then filled with amalgam, and fitted a crown. It had produced no irritation.

Dr. Gaskill stated that he had had a case three years ago similar to the one shown by Dr. Boice, and that it had produced no trouble.

Dr. Pierce, in answer to Dr. Truman's remarks, said, "In speaking of nature's toleration I had reference to the different conditions of patients and the success that was attained when working under adverse circumstances. My surprise has been that nature so readily tolerates the application of crowns or caps or collars on the roots of teeth. Of all placed in the mouth, there are very few that have the adaptation of those exhibited here to-night, and yet a large proportion are worn for years with great comfort, and they are tolerated by nature, though not fitting accurately. There are hundreds of caps and collars put in that do not have the adaptation described by Dr. Kirk. The irritation that we have usually caused by the collar and cap is as much from the improper condition of the root as from the fact that it wears a collar. Although the operation is not a complete success, so far as workmanship is concerned, yet the absence of fit is not always the source of irritation."

*Dr. Bonwill.*—I ought not to say one word on crowns after I have spoken so often on the subject. Every one knows that I am opposed upon principle to a collar being placed upon a tooth. I cannot see where strength is increased, yet some claim there is, and that it prevents the cement used to secure it from becoming injured. My experience began in 1873, when I first put on a crown independent of a wood pivot. I went to all extremes in taking anything and everything offered me. I wanted to see what was the farthest extent I could go to. If we examine the teeth that are to-day pivoted by the average dentists, we find there is very little decay in the root of the tooth. So far as injury to the pericemental membrane is concerned, I have a tooth here treated by a dentist in the country. When it came to me it was in a general state of ulceration from the apex. You can see the condition in which the band was placed in the mouth. The pericemental membrane was entirely gone. I suppose the cause of it was the collar. I know that for ten years before the tooth had had a gold filling in it. The gentleman was in the country when the tooth troubled him, and he went to the nearest dentist, who placed this crown, and the consequence was its entire destruction. Without a collar it would have been in his mouth to-day. I have one here I placed in fifteen years ago with a nut and screw. Finally, a large part of it broke away. I filled it in with amalgam, and had no idea it was so rough until it came out. It lasted for seven years in that condition. Judging from the number of cases I have seen treated in the hands of others, I see no reason to change my views. There are a few cases where I would put on a collar, but would cement it with a pin in the root of the tooth, then I would have something by which I could hold it, and it could not move; but to depend on pushing a crown with a band on it with ordinary cement, and expect that collar to sustain the root, it will not do it. If you put it up high it will produce inflammation that will destroy the tooth. So my experience has been against collars, and especially the manner in which they are placed.

The specimens exhibited here to-night show that any one who knows how and where to cut can do it, but it cannot be done in the mouth. (Dr. Kirk, in answer to a question of Dr. Bonwill's, replied that the work was not done in the mouth.) There is no use of bringing such samples here and expecting us to do it. They are entirely misleading. Dr. Kirk does not expect to make such successes as that. I object to so much gold upon the cutting edges and upon the grinding surface of the tooth as you are compelled to put to keep them from being fractured.

